# Effects of a Valerian‐Hops Extract Combination (Ze 91019) on Sleep Duration and Daytime Cognitive and Psychological Parameters in Occasional Insomnia: A Randomized Controlled Feasibility Trial

**DOI:** 10.1002/brb3.70600

**Published:** 2025-06-04

**Authors:** Nathalie Schicktanz, Christiane Gerhards, Thomas Schlitt, Amanda Aerni, Elia Müggler, Dominique de Quervain, Andreas Papassotiropoulos, Georg Boonen, Juergen Drewe, Veronika Butterweck

**Affiliations:** ^1^ Division of Cognitive Neuroscience, Department of Biomedicine University of Basel Basel Switzerland; ^2^ Research Cluster Molecular and Cognitive Neurosciences, Department of Biomedicine University of Basel Basel Switzerland; ^3^ Division of Molecular Neuroscience, Department of Biomedicine University of Basel Basel Switzerland; ^4^ Psychiatric University Clinics University of Basel Basel Switzerland; ^5^ Max Zeller Soehne AG Medical Department Romanshorn Switzerland

**Keywords:** cognition, feasibility study, fitbit charge 5, herbal medicine, hops, redormin, sleep, valerian, Ze 91019

## Abstract

**Objective/Background:**

This study evaluated the feasibility of investigating the effect of a valerian and hops‐based herbal medication (Ze 91019) on daytime cognitive performance, psychological parameters, and sleep measures in individuals with occasional sleep problems.

**Methods:**

A randomized, double‐blind, placebo‐controlled study was conducted in 40 participants over a 21‐day run‐in period and a 21‐day treatment period. Participants used Fitbit sleep trackers and completed daily online cognitive tests (i.e., reaction time and working memory), and surveys to assess subjective psychological outcomes (i.e., cognitive performance, stress levels, tiredness, mood, quality of life, and motivation).

**Results:**

The study design proved feasible, with high adherence to the study protocol. Exploratory analyses revealed a statistically significant increase in sleep duration during the treatment period for participants using Ze 91019 compared to placebo (mean daily increase: 21.7 min, *p* = 0.019) without statistically significant effects on cognitive or psychological outcomes. Moreover, Ze 91019 statistically significantly increased the sleep duration of the shortest night in the treatment period by 48.7 minutes. The medication was well‐tolerated.

**Conclusions:**

The study design proved feasible, and Ze 91019 increased sleep duration without affecting daytime cognitive or psychological outcomes.

**Trial Registration:**

The trial has been preregistered at www.clinicaltrials.gov (NCT05684523).

## Graphical abstract

Valerian‐hops extract combination (Ze 91019) significantly increased sleep duration in individuals with occasional sleep problems by 21.7 minutes per night and 48.7 minutes on the shortest night, compared to placebo, without impairing daytime cognitive or psychological functioning. This randomized controlled trial demonstrated high adherence and proved feasibility.

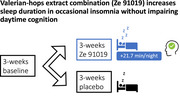

## 1 Introduction

Sleep disturbances affect a significant portion of the adult general population, with about 30% reporting insomnia symptoms (Morin et al. 2006). Ze 91019 is an herbal extract combining valerian root (*Valeriana officinalis*) and hop cones (*Humulus lupulus*), traditionally used to address sleep disturbances. This specific formulation is available in Switzerland and several other countries. Ze 91019 has shown central effects in rats by increasing the spectral power in the delta, theta, and alpha frequency bands of the frontal cortex (Dimpfel et al. 2006). In addition, in human studies, Ze 91019 has been shown to counteract central arousal induced by 200 mg of orally administered caffeine (Schellenberg et al. 2004). Furthermore, a randomized controlled trial conducted over four weeks reported a significant reduction in sleep latency for patients with non‐organic sleep disorders characterized by prolonged sleep latency (Koetter et al. 2007). However, despite the promising sleep benefits of valerian‐hops extracts, no studies have yet explored their potential impact on daytime cognitive performance. This gap is particularly important since many conventional hypnotics, including widely prescribed benzodiazepines, are known to negatively affect cognition, particularly in areas like attention and memory (Buffett‐Jerrott and Stewart 2002). This study aimed to evaluate the feasibility of investigating the effects of Ze 91019 on daytime cognitive performance, psychological well‐being, and sleep parameters, as measured by a sleep tracker, in healthy individuals experiencing occasional sleep problems. Evaluating the next‐day residual effects, such as psychomotor impairment or sedation, is essential for determining whether a sleep medication could pose risks in activities like driving, operating machinery, or other tasks requiring alertness.

## 2 Methods

### 2.1 Study Design

This was a randomized, double‐blind, placebo‐controlled study conducted at the University of Basel, Switzerland. The study involved a 21‐day run‐in period followed by a 21‐day treatment period. Participants were randomized to receive either Ze 91019 or a matching placebo. Daily cognitive performance and psychological parameters were measured online (see below), and sleep parameters and heart rate were assessed using Fitbit sleep trackers.

### 2.2 Participants

The inclusion criteria for this study required participants to be healthy males or females between the ages of 18 and 65. Eligible individuals needed to experience occasional sleep problems, defined as sleep disturbances occurring on average 1–2 nights per week, with a Pittsburgh sleep quality index (PSQI) (Buysee et al. 1989) score greater than 5. In addition to sleep issues, participants were also required to report subjective cognitive difficulties, occurring at least once a week on average over the past month. Participants were excluded if they had a DSM‐V diagnosis of insomnia, as determined by the mini‐DIPS (Margraf 1994). Participants were also excluded if they were currently taking prescription medications with psychotropic effects or over‐the‐counter medications for sleep or mood problems. Participants gave written informed consent before enrollment.

As this was a feasibility study, no formal power calculation was conducted to estimate the required sample size. Instead, we aimed to include 40 participants, a figure aligned with the commonly recommended range for pilot and feasibility studies (Whitehead et al. 2016).

### 2.3 Study Medication/Placebo

Redormin 500 contains as active substance 500 mg of Valerianae radix (*Valeriana officinalis* L.) dry extract, DER 4–6:1, extraction solvent methanol 45% m/m, and 120 mg of Lupuli flos (*Humulus lupulus* L.) dry extract, DER 5–7:1, extraction solvent methanol 45% m/m (extract code Ze 91019). Placebo medication was identical in presentation, shape, and color, and similar in scent to Redormin 500. One film‐coated tablet of Redormin 500 or placebo was administered about 1 h before bedtime with some liquid for 21 days.

### 2.4 Outcome Measures

The primary outcome was the feasibility of the study design, measured by recruitment success, adherence to the study protocol, and technical issues. Adherence was monitored through daily online surveys and Fitbit data. Secondary outcomes included sleep parameters, cognitive performance, and subjective psychological parameters. Sleep parameters were measured by the sleep tracker (Fitbit Charge 5). A validation study using the previous version of the Fitbit Charge 5 used in the present study, i.e., Fitbit Charge 4, exhibited overall robust performance in measuring sleep duration when compared to polysomnography (Dong 2022). Sleep onset latency could not be assessed within the home setting of the study, and sleep stage data were not reported due to concerns about the accuracy of consumer‐grade trackers (Dong 2022). Cognitive performance [i.e., reaction time assessed with the red button task (Dinges and Powell 1985) and working memory assessed with the digit span backwards (Aster et al. 2006)], and subjective psychological parameters (using visual analog scales for cognitive performance, stress, tiredness, mood, motivation, sleep quality and quality of life) were assessed via daily online surveys with SoSci‐Survey (Version 3.5.01, https://www.soscisurvey.de, server supported by sciCORE https://scicore.unibas.ch scientific computing core facility at University of Basel) in the run‐in and treatment periods between 5 pm and 12 pm (see Supporting Information for details). Safety was measured by recording adverse events.

### 2.5 Statistical Analysis

Descriptive statistics were used to assess feasibility outcomes. For secondary outcomes, treatment effects were analyzed with parametric and non‐parametric statistics (the latter when the assumption of normality was not met) using the means of the secondary outcomes over the treatment period as dependent variables. Age, sex, and baseline (i.e. means of the secondary outcomes measured over the last 7 days during the run‐in period) were included as covariates. Furthermore, we investigated the treatment effects during and after the shortest night with age, sex, and baseline (i.e. secondary outcomes measured during the shortest night (for sleep‐related parameters) and the following day (for cognitive and psychological parameters) during the run‐in period) as covariates. Lastly, we assessed whether cognitive performance following the shortest night during the run‐in period differed from performance after all other days in the same period. The software environment R was used for statistical analyses (version 4.3.2, RStudio 2022). For details, see Supporting Information.

## 3 Results

### 3.1 Feasibility

**FIGURE 1:**
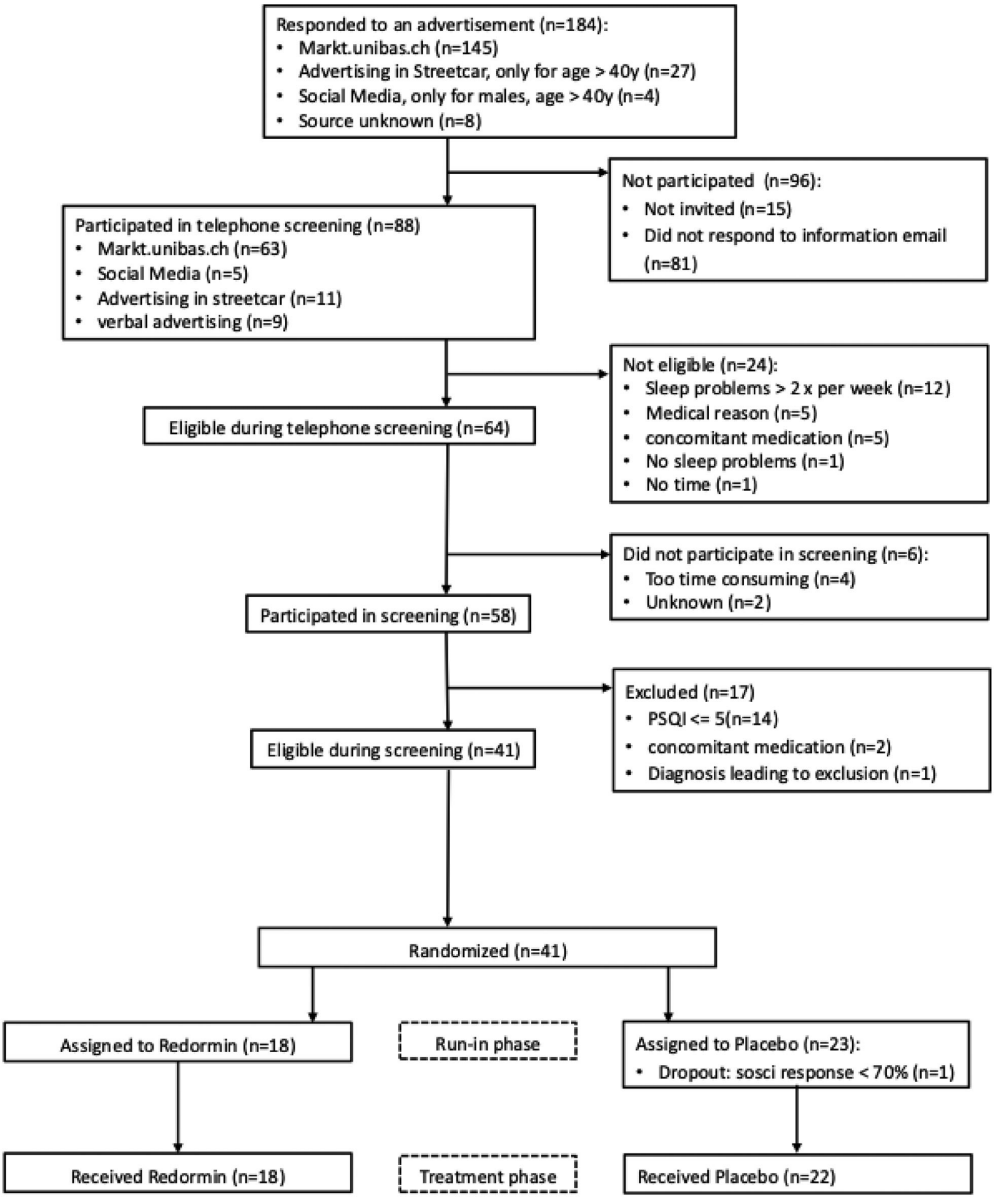
Study Flow Chart.

Of the 184 subjects interested in the study, 88 participated in the telephone screening. Around 58 subjects participated in the screening from which 17 were excluded [PSQI score ≤ 5 (*N* = 14), concomitant medication (*N* = 2), diagnosis leading to exclusion (*N* = 1)]. Consequently, 41 subjects were randomized (18 to Ze 91019, 23 to placebo), and 40 subjects (18 in Ze 91019, 22 in placebo group) completed the trial. One subject dropped out because adherence to the use of the online tool SoSci‐Survey was less than 70% (defined in the protocol as the threshold for exclusion) in the run‐in period (see Figure 1).

For the included subjects (*N* = 40), adherence to the use of the online tool SoSci‐Survey was >89% (mean) of test days, adherence to study medication was >89% (mean) of test days and adherence to the use of the Fitbit tracker was >96% (mean) of test days.

There were some technical problems with the Fitbit tracker and SoSci‐Survey, which were mostly resolved during the study and generally resulted in minimal missing data. However, an exception was the HRV data, which was at least partially missing for 14 subjects due to a setting issue within the Fitbit app. Hence, no statistical analysis was performed for HRV.

Qualitative feedback on the study: While all 40 subjects provided positive feedback on the study, 22 out of 40 (11 verum, 11 placebo) also offered negative feedback, most frequently citing the effort required for the daily cognitive tasks as a hassle (8 mentions: 4 verum, 4 placebo).

Belief having received verum or placebo: Of the subjects who received placebo (*N* = 22), 16 subjects believed they had received placebo and 6 believed they had received verum. Of the subjects who received verum (*N* = 18), 5 subjects believed they had received a placebo and 13 believed they had received verum. A chi‐square test revealed that the correct allocation was above the chance level (*p* = 0.01). The qualitative feedback on the reasons for their belief indicated that, among participants who correctly identified their allocation based on a single reason, the absence or presence of an effect accounted for 87.0% of correct guesses (20 participants: 15 from the placebo group and 5 from the verum group). In comparison, the absence or presence of a characteristic smell accounted for 13.0% of correct guesses (3 participants: all from the verum group).

For demographics of the treatment and verum groups, see Table 1.

**TABLE 1:** **Demographics**. Fisher's exact test was applied for sex, while an independent *t*‐test was used for all other variables to compare the assignment groups.

	Placebo (*N* = 22) mean (SD), range	Ze 91019 (*N* = 18) mean (SD), range	*p*
Sex (f/m)	12/10	10/8	1
Age	36.55 (14.46), 18–61	36.56 (14.22), 19–60	1
Bodyweight (kg)	73.85 (14.61), 54.2–103.0	72.26 (14.03), 50.3–97.1	0.73
Body mass index (BMI)	24.75 (3.34), 19.0–33.8	24.08 (2.99), 18.8–29.4	0.51
PSQI score at screening	7.95 (1.91), 6–11	9 (2.14), 6–13	0.12

### 3.2 Sleep Duration, Cognitive Performance, and Subjective Psychological Outcomes

#### 3.2.1 Entire Treatment Period

**FIGURE 2:**
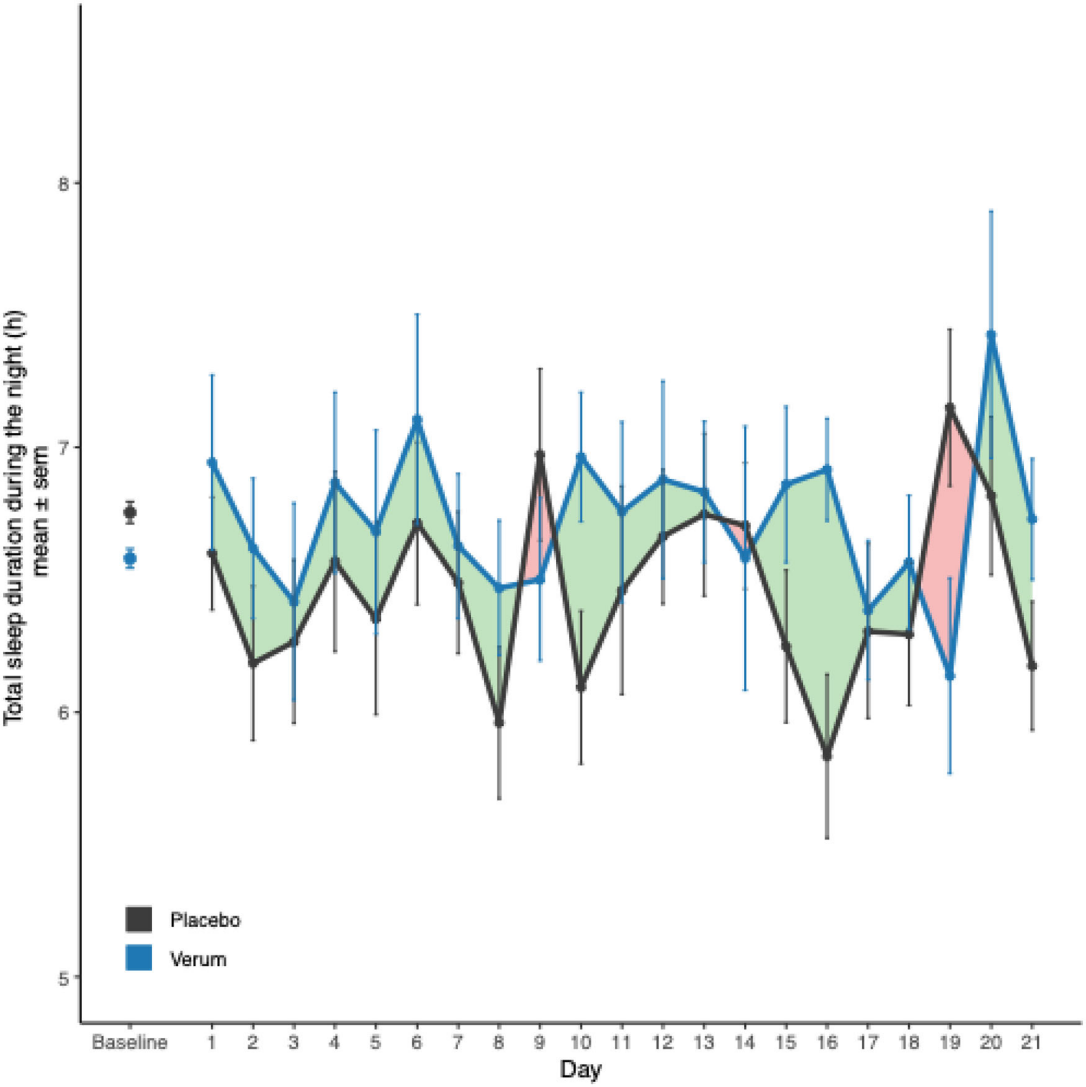
**Treatment effect on total sleep duration**. Presented are raw, uncorrected means over the treatment period with standard errors of the mean. Baseline refers to the baseline value used in the statistical model (i.e., the mean of total sleep duration measured over the last 7 days in the run‐in period). The green colored areas illustrate an increase in sleep duration under verum compared to placebo. The red colored areas illustrate an increase in sleep duration under placebo compared to verum.

Ze 91019 significantly increased sleep duration compared to placebo, with an average increase of 0.362 h (21.7 min) per night during the treatment period (*p* = 0.019, *r* = 0.38, Figure 2, Table 2). Importantly, the treatment effect was not driven by the correct allocation of the treatment that was above the chance level [interaction effect: treatment x correct allocation (yes/no); *p* = 0.49]. We also found a significant prolongation of the total duration in bed during the night (*p* = 0.023, *r* = 0.37) with an estimated increase in the duration in bed of 0.406 h (24.4 min) in the verum group compared to the placebo group (Table 2). Further, a significant improvement of sleep was indicated in the overall sleep score (a Fitbit variable composed of the following components: sleep duration, sleep quality, and restoration, for a total score of up to 100) (*p* = 0.03, *r* = 0.36) with an estimated increase in the score of 2.14 points in the verum group compared to the placebo group (Table 2). No significant treatment effects were observed for cognitive performance (working memory: *p* = 0.27; reaction time: *p* = 0.44), subjective psychological parameters, or heart rate (*p* ≥ 0.26, Table 2).

**TABLE 2:** **Treatment effects on sleep parameters, day‐time cognitive performance, subjective psychological outcomes and heart rate**. The outcome values presented are raw, uncorrected means over the treatment period with standard deviations in parentheses. For the entire treatment period, baseline values correspond to nighttime parameters (e.g., sleep‐related outcomes) and daytime parameters (e.g., cognitive and psychological outcomes) measured as the means over the last 7 days of the run‐in period. For the shortest night, baseline values correspond to outcome variables measured during the shortest night (for nighttime parameters) and the following day (for daytime variables) during the run‐in period. The *p*‐values are derived from a statistical model that adjusted for age, sex, and baseline values (see Supporting Information for details). Visual analog scale (VAS) scores range from 1 to 101, with higher values indicating stronger subjective intensity.

Entire treatment period				
Outcome	Period	Placebo	Ze 91019	*p*
Total sleep duration during the night (*h*)	Baseline	6.76 (0.88)	6.61 (0.69)	
	Treatment	6.46 (0.74)	6.73 (0.53)	0.019
Total duration in bed during the night (*h*)	Baseline	7.78 (1.10)	7.61 (0.89)	
	Treatment	7.40 (0.84)	7.72 (0.67)	0.023
Fitbit sleep score (points)	Baseline	76.20 (4.29)	74.84 (3.07)	
	Treatment	74.94 (4.95)	76.07 (3.20)	0.03
Working memory (correct responses)	Baseline	8.98 (2.43)	8.61 (2.2)	
	Treatment	9.06 (2.49)	9.03 (2.17)	0.27
Reaction time (correct responses)	Baseline	377.92 (102.98)	403.74 (93.91)	
	Treatment	378.38 (110.45)	401.59 (84.7)	0.44
Subjective cognitive performance (VAS)	Baseline	60.61 (10.00)	57.83 (19.10)	
	Treatment	60.09 (8.22)	61.54 (14.38)	0.33
Tiredness (VAS)	Baseline	54.33 (14.57)	59.29 (14.92)	
	Treatment	52.58 (11.98)	53.23 (10.67)	0.57
Mood (VAS)	Baseline	63.22 (8.49)	59.15 (16.35)	
	Treatment	63.72 (7.75)	62.39 (13.40)	0.91
Stress (VAS)	Baseline	51.23 (15.2)	51.13 (16.74)	
	Treatment	48.82 (15.8)	48.31 (17.26)	0.89
Motivation (VAS)	Baseline	55.97 (9.69)	55.17 (15.73)	
	Treatment	56.97 (9.68)	58.65 (12.38)	0.51
Quality of life (VAS)	Baseline	59.24 (8.83)	58.94 (15.44)	
	Treatment	59.97 (6.88)	62.78 (13.41)	0.27
Sleep quality (VAS)	Baseline	58.85 (11.89)	52.68 (11.31)	
	Treatment	57.46 (9.18)	54.8 (8.28)	0.57
Resting heart rate during night (bpm)	Baseline	61.59 (7.05)	61.85 (8.10)	
	Treatment	61.35 (6.40)	62.02 (7.06)	0.53

Shortest night				
Outcome	Period	Placebo	Ze 91019	*p*
Total sleep duration during the night (*h*)	Baseline	4.30 (1.06)	3.97 (1.22)	
	Treatment	3.89 (1.08)	4.51 (1.33)	0.016
Total duration in bed during the night (*h*)	Baseline	4.85 (1.26)	4.57 (1.44)	
	Treatment	4.41 (1.23)	5.25 (1.59)	0.015
Fitbit sleep score (points)	Baseline	67.78 (5.39)	64.29 (5.89)	
	Treatment	67.12 (7.42)	66.13 (6.54)	0.94
Working memory (correct responses)	Baseline	8.65 (3.05)	8.5 (2.03)	
	Treatment	8.95 (3.09)	8.27 (3.15)	0.48
Reaction time (correct responses)	Baseline	355.63 (97.81)	419.09 (140.79)	
	Treatment	390.13 (136.46)	445.73 (155.92)	0.24
Subjective cognitive performance (VAS)	Baseline	45.6 (15.33)	44.69 (26.26)	
	Treatment	58.2 (24.77)	49.13 (24.22)	0.41
Tiredness (VAS)	Baseline	76.4 (18.19)	72.19 (24.02)	
	Treatment	64.9 (27.91)	73.73 (18.48)	0.24
Mood (VAS)	Baseline	55.85 (20.32)	55 (16.13)	
	Treatment	62.35 (19.52)	49.53 (19.95)	0.15
Stress (VAS)	Baseline	58.55 (15.20)	55.00 (21.92)	
	Treatment	41.00 (23.92)	49.80 (28.97)	0.25
Motivation (VAS)	Baseline	49.70 (17.08)	44.69 (17.79)	
	Treatment	42.15 (28.26)	45.93 (26.26)	0.66
Quality of life (VAS)	Baseline	49.45 (18.77)	50.12 (19.35)	
	Treatment	50.30 (19.74)	56.33 (25.25)	0.52
Sleep quality (VAS)	Baseline	29.55 (23.16)	28.19 (27.22)	
	Treatment	37.25 (25.38)	33.53 (26.99)	0.99
Resting heart rate during night (bpm)	Baseline	60.5 (6.20)	62.5 (8.27)	
	Treatment	59.65 (5.73)	63.06 (8.51)	0.77

#### 3.2.2 Shortest Night

Ze 91019, as compared to placebo, significantly increased sleep duration of the shortest night in the treatment period by 0.812 h (48.7 min; *p* = 0.016, *r* = 0.39; Table 2). Again, this treatment effect was not driven by the correct allocation of the treatment that was above the chance level (interaction effect: treatment x correct allocation (yes/no); *p* = 0.71). Consistent with the prolongation of total sleep duration in the shortest night, a nominally significant effect was also seen for the total duration in bed during the night (*p* = 0.015, *r* = 0.40) with an estimated increase in time in bed of 0.99 h (59 min) in the verum group compared to the placebo group (Table 2).

However, Ze 91019 as compared to placebo did not significantly affect day time objective cognitive performance (reaction time, working memory), subjective psychological parameters, or heart rate on the day after the shortest night in the treatment period (*p* ≥ 0.14; Table 2).

Since cognitive deficits were most likely to occur following the shortest nights, we investigated whether daytime cognitive performance after the shortest night during the run‐in period differed from performance on all other days in this period, independent of treatment effects. No significant differences were observed on working memory or reaction time (*p* ≥ 0.13), but on subjective psychological parameters (cognitive performance, stress, tiredness, mood, motivation, sleep quality, and quality of life, all *p* < 0.026).

#### 3.2.3 Safety

A total of 29 adverse events (AEs) occurred during the study in 18 participants; 14 AEs in the run‐in period, 14 in the treatment period, and 1 between periods. During the treatment period, 5 AEs occurred in the placebo group, 9 AEs in the verum group (see Table S1 for details). There was no significant association between the treatment groups and the occurrence of AEs during the treatment period (Fisher exact test, *p* = 0.11). All AEs were mild to moderate. No serious AEs occurred during the study.

## 4 Discussion

Overall, the study design proved feasible with high adherence rates and manageable technical issues. Ze 91019 was generally well tolerated.

The exploratory analysis indicated that the valerian and hop‐fixed herbal combination (Ze 91019) significantly increased sleep duration compared to placebo, with an average increase of 21.7 min per night during the treatment period as measured by the Fitbit tracker. Moreover, Ze 91019 significantly increased the sleep duration of the shortest night in the treatment period by 48.7 minutes. This substantial increase highlights the potential of Ze 91019 to improve sleep duration under conditions of restricted sleep.

Ze 91019 did not have a significant impact on daytime objective or subjective cognitive performance or psychological parameters, neither over the entire treatment period nor specifically after the shortest night, at the given study power. This lack of significant findings in objective and subjective cognitive and psychological outcomes suggests that while Ze 91019 can improve sleep duration, it does not translate to noticeable improvements in daytime cognitive or emotional functioning within the scope of this feasibility study. On the other hand, it is important to note that Ze 91019 did not lead to a significant worsening of objective or subjective cognitive performance, as is often the case with several other pharmacological sleeping aids, in particular benzodiazepines (Buffett‐Jerrott and Stewart 2002). In addition, we found in the run‐in period that objective cognitive measures after the shortest night were comparable to those after all other nights, suggesting that cognitive performance was not substantially affected by variations in sleep duration in this population with occasional sleep problems.

The absence of significant next‐day effects in our study does not necessarily preclude the potential cognitive benefits of Ze 91019. It is possible that the cognitive tasks employed were not sensitive enough to detect subtle changes, or that the relatively short duration of the study limited the ability to observe cumulative improvements. Notably, even small differences in habitual sleep duration may have meaningful cognitive implications. For example, a recent large‐scale study in adolescents reported that just a 15‐min increase in average nightly sleep duration was associated with significantly better cognitive performance across several domains (Ma et al. 2025). This highlights the potential for modest improvements in sleep to yield measurable cognitive benefits over time, which may become more apparent with longer treatment periods or more comprehensive cognitive tests.

While adherence to the online assessment was above 89%, eight participants noted the effort required for the daily cognitive tasks. In future studies with longer durations and daily assessments, it will be important to account for adherence in sample size calculations, as well as to consider the frequency, duration, and type of cognitive tasks, potentially including motivational elements to support engagement.

Participants were able to guess their treatment allocation above a chance level, raising the possibility of expectancy effects. However, correct guesses were primarily driven by perceived effects rather than by sensory cues of the intervention. Furthermore, there was no indication that these guesses influenced the observed outcomes: the interactions between treatment effects and guess accuracy were non‐significant (*p* = 0.49 and *p* = 0.71 for the entire treatment period and the shortest night, respectively), making it unlikely that the observed treatment effects on sleep duration were driven by expectancy bias.

In conclusion, the study design proved feasible, and we found evidence suggesting that Ze 91019 increases sleep duration as measured with the Fitbit tracker without significantly affecting daytime objective or subjective cognitive performance or other psychological outcomes.

## Author Contributions

**Nathalie Schicktanz**: validation, visualization, writing–review and editing, software, formal analysis, writing–original draft, methodology. **Christiane Gerhards**: investigation, writing–review and editing, project administration. **Thomas Schlitt**: data curation, validation, writing–review and editing. **Amanda Aerni**: investigation, writing–review and editing, project administration. **Elia Müggler**: investigation, writing–review and editing, data curation. **Dominique de Quervain**: investigation, conceptualization, funding acquisition, writing–original draft, methodology, writing–review and editing, resources. **Andreas Papassotiropoulos**: conceptualization, investigation, funding acquisition, writing–review and editing, resources, methodology. **Georg Boonen**: conceptualization, writing–review and editing, resources, supervision. **Juergen Drewe**: conceptualization, writing–review and editing, resources, supervision. **Veronika Butterweck**: conceptualization, writing–review and editing, resources, supervision, writing–original draft.

## Ethics Statement

The study protocol has been approved by the Ethics Committee of Northwestern and Central Switzerland before the start of the trial.

## Consent

All participants gave written informed consent before enrollment.

### Peer Review

The peer review history for this article is available at https://publons.com/publon/10.1002/brb3.70600

## Supporting information



Table S1. Overview of AEs reported during the treatment phase, stratified by verum and placebo groups, along with the investigator's assessment of their relatedness to the study medication.

## Data Availability

The data supporting the findings of this study can be made available upon reasonable request to the corresponding authors.
