# Supplementary material for: Effects of a Valerian‐Hops Extract Combination (Ze 91019) on Sleep Duration and Daytime Cognitive and Psychological Parameters in Occasional Insomnia: A Randomized Controlled Feasibility Trial
Source: Brain Behav. 2025 Jun 4;15(6):e70600. doi: 10.1002/brb3.70600 (PMC12134488; doi:10.1002/brb3.70600)
Supplement: Supplementary file 1 — Table S1. Overview of AEs reported during the treatment phase, stratified by verum and placebo groups, along with the investigator's assessment of their relatedness to the study medication. [file BRB3-15-e70600-s001.docx]

**Supplementary Information**

### Assessment of secondary outcomes

Diary with SoSci Survey Questionnaire (run-in period and treatment period)

*Cognitive parameters*

- *Reaction time test (Red Button Task)*, adapted from Dinges and Powell (1985): The participant is presented a gray circle (button) in the middle of the screen. The participant is instructed to push this button as soon as its color changes to red. The test runs for 2 minutes, with the intervals between color changes randomly varying between 2 and 5 seconds.

*Score*: The average of all reaction times over 2 min. The period between the time of color change and button push is measured in milliseconds and is considered as reaction time.

- *Working memory* was assessed with the digit span task backward, a subtest of the “Wechsler Intelligenztest für Erwachsene“ (WIE; (von Aster 2006)). Sequences of digits ranging from 0 to 9 are visually presented on a screen, with each digit appearing for 1 second before being replaced by the next. Each digit appears only once in a given sequence. After the entire sequence has been displayed, the participant is prompted to recall the digits in reverse order. For example, if the screen displays "4, 7, 2" sequentially, the correct response would be "2, 7, 4." The length of the digit sequences starts at two digits and increases incrementally as the participant successfully completes each level. To prevent repetition, sequences of the same length are always distinct.

*Stop rule*: both digit spans of a level (same length) were wrongly reproduced

*Score*: number of correct remembered digit spans. Total scores for digit span backward will be calculated as described in the manual of the WIE

*Psychological parameters (Visual Analog Scales, VAS):*

Visual analog scales (VAS) were used to assess the psychological parameters and the sleep quality during the run-in period and the treatment period. Participants specified their level of agreement to the statement by indicating a position along a continuous line between two end-points. The score ranges from 1 to 101.

Endpoints of the VAS for

- Subjective cognitive performance during the day: very poor and very good
- Tiredness during the day: not at all and very strong
- Mood during the day: very bad and very good
- Stress level during the day: very low and very high
- Motivation during the day: not at all and very high
- Quality of life (regarding cognitive performance) during the day: very low and very high
- Sleep quality last night: very bad and very good

These parameters were assessed daily using online surveys with SoSci-Survey (Version 3.5.01, https://www.soscisurvey.de, server supported by sciCORE https://scicore.unibas.ch scientific computing core facility at University of Basel) in the run-in and treatment periods between 5 pm and 12 pm.

Objective sleep parameters, heart rate variability (HRV) and heart rate (HR) collected with sleep tracker Fitbit (run-in period and treatment period)

We used Fitbit Charge 5 devices. When the Fitbit Charge 5 device is worn by the participant, it tracks sleep data in addition to other activity data automatically. The data was collected via the Fitbit app on the users’ smartphone and then collated at the servers of the company when the Fitbit device was connected via Bluetooth to the smartphone. For each participant data was downloaded from Fitbit to University of Basel servers by data requests at visit 1 (technical checks), visit 2 (to check compliance with minimum time of wearing the Fitbit device) and visit 3 (final data acquisition for statistical analyses). Only data needed for data analyses for this study were retained as described in the study protocol.

***Statistical methods for exploratory analyses of secondary variables***

Entire treatment period: Treatment effects over the entire treatment period were analyzed using the means of the secondary outcomes over the entire treatment period, with age, sex, and baseline (i.e. means of the dependent variables over the last 7 days in the run-in phase) as covariates.

Model: dependent variable ~ sex + age + baseline + treatment (placebo or verum)

Shortest night: Treatment effects during/after the shortest night were analyzed using the secondary outcome during/after the shortest night during the treatment period, with age, sex, and baseline (i.e. secondary outcomes measured during the shortest night (for sleep-related parameters) and the following day (for cognitive and psychological parameters) during the run-in period) as covariates.

Model: dependent variable ~ sex + age + baseline + treatment (placebo or verum)

We used both parametric (linear) models and non-parametric models (Mann-Whitney U test); the latter when normality assumption was not met. The assumption of normally distributed residuals was tested using the *check_normality* function from the *performance* library, specifically with the Shapiro-Wilk test. For non-parametric models, the dependent variable was residualized for age, sex, and baseline.

The software environment «R» was used for statistical computing (version 4.3.2, Rstudio 2022) with the following libraries: Statistics: *lme4, r2glmm (*parametric*)*; *rstatix (*non-parametric*), performance (*normality*);* Graphics: *ggplot2, dplyr.*

Adverse events

Table S1. Overview of adverse events reported during the treatment phase, stratified by verum and placebo groups, along with the investigator’s assessment of their relatedness to the study medication.

|  | Verum | Placebo | Relatedness |
| --- | --- | --- | --- |
| Headache | 1 | 2 | All unlikely |
| Viral Rhinitis | 2 | 2 | Verum: 1 x unlikely; 1 x not related  Placebo: 2 x unlikely |
| Flu-like infection | 1 | 0 | Unlikely |
| Covid-19 infection | 1 | 0 | Not related |
| Diarrhea | 1 | 0 | Unlikely |
| Bladder irritation | 1 | 0 | Not related |
| Skin irritation (from Fitbit) | 2 | 1 | All not related |
| **Sum** | **9** | **5** |  |

**References**

Dinges, D.F., Powell, J.W. Microcomputer analyses of performance on a portable, simple visual RT task during sustained operations. Behavior Research Methods, Instruments, & Computers 17, 652–655 (1985).

von Aster M, Neubauer, A., & Horn, R. . Wechsler Intelligenztest für Erwachsene (WIE). Deutschsprachige Bearbeitung und Adaptiation des WAIS-III von David Wechseler. *Frankfurt, Harcourt Test Services* 2006.

R Core Team (2022) R: A Language and Environment for Statistical Computing. R Foundation for Statistical Computing, Vienna.
https://www.R-project.org
